# Is Sleep Essential for Neural Plasticity in Humans, and How Does It Affect Motor and Cognitive Recovery?

**DOI:** 10.1155/2013/103949

**Published:** 2013-06-11

**Authors:** Maurizio Gorgoni, Aurora D'Atri, Giulia Lauri, Paolo Maria Rossini, Fabio Ferlazzo, Luigi De Gennaro

**Affiliations:** ^1^Department of Psychology, “Sapienza” University of Rome, 00185 Rome, Italy; ^2^Institute of Neurology, Catholic University of the Sacred Heart, 00168 Rome, Italy; ^3^IRCCS San Raffaele Pisana, 00163 Rome, Italy; ^4^IRCCS Fondazione Santa Lucia, 00179 Rome, Italy

## Abstract

There is a general consensus that sleep is strictly linked to memory, learning, and, in general, to the mechanisms of neural plasticity, and that this link may directly affect recovery processes. In fact, a coherent pattern of empirical findings points to beneficial effect of sleep on learning and plastic processes, and changes in synaptic plasticity during wakefulness induce coherent modifications in EEG slow wave cortical topography during subsequent sleep. However, the specific nature of the relation between sleep and synaptic plasticity is not clear yet. We reported findings in line with two models conflicting with respect to the underlying mechanisms, that is, the “synaptic homeostasis hypothesis” and the “consolidation” hypothesis, and some recent results that may reconcile them. Independently from the specific mechanisms involved, sleep loss is associated with detrimental effects on plastic processes at a molecular and electrophysiological level. Finally, we reviewed growing evidence supporting the notion that plasticity-dependent recovery could be improved managing sleep quality, while monitoring EEG during sleep may help to explain how specific rehabilitative paradigms work. We conclude that a better understanding of the sleep-plasticity link could be crucial from a rehabilitative point of view.

## 1. Introduction

In 1971, Rechtschaffen [1] stated that “if sleep does not serve an absolute vital function, then it is the biggest mistake the evolutionary process ever made….” Indeed, almost all the animal species, from the largest mammals to the fruit flies [2], show a behavioral state that can be considered sleep-like. Sleep seems to be a crucial need, as much as drinking or eating, to such an extent that chronic sleep deprivation in rats produces cellular and molecular changes in brain [3] that makes the animal die within a matter of weeks [4].

Different hypotheses were suggested to explain the functions of sleep, but a general consensus exists today that sleep is strictly linked to memory, learning and, in general, to the mechanisms of neural plasticity. Indeed, cognitive impairments, especially in learning, and memory tasks [5–7], are one of the main consequences of sleep deprivation. Although the link between sleep, memory, and neural plasticity has been widely investigated, such a relation is not yet completely understood. Several findings are in line with the hypothesis of a homeostatic, sleep-mediated synaptic downregulation [8, 9], while other studies support a “consolidation” model based on the reactivation, during sleep, of the same areas which were active during wakefulness [10, 11]. Since it is widely accepted that synaptic plasticity mechanisms underlie motor and cognitive recovery, understanding the relationship between sleep and plasticity is essential also in a rehabilitation perspective. 

The main aim of the present paper is to review studies showing how sleep-dependent plasticity could be involved in functional recovery from different neuropsychological conditions (poststroke brain damage, obstructive sleep apnea, Alzheimer's disease, and autism) and to provide insights on how the efficacy of rehabilitation protocols could be improved by methods which enhance sleep-dependent plasticity. The term “functional recovery” has different implications for the various clinical conditions considered, depending on the hypothetical mechanisms underlying each disturb. In the current review, we will adopt a very general definition and will refer to it as a process involving improvement or slowing down of deterioration in different areas (motor and/or cognitive) of the illness. Aiming to propose a possible role of sleep-dependent plasticity in functional recovery, we will shortly summarize how sleep and plastic process are related. Namely, we will discuss the role of sleep in ensuring the consolidation of plastic changes and the possibility of learning new things every day. To this aim, we will highlight the findings about how plastic changes during wakefulness affect the subsequent sleep and try to understand what kind of plastic modifications occurs during sleep. Then, we will report empirical evidence about the molecular and electrophysiological consequences of sleep deprivation on the plastic mechanisms. Finally, we will discuss how sleep-dependent plasticity could influence functional recovery, and we will suggest how to increase efficacy of rehabilitative protocols by enhancing sleep quality, reducing sleep disorders, and promoting sleep-dependent plasticity. 

## 2. Influence of Plastic Changes during Wakefulness on Subsequent Sleep

Spontaneous wakefulness is characterized by molecular changes associated with long-term potentiation (LTP) [12, 13], and by an increase of synaptic density [14]. The synaptic homeostasis hypothesis [8, 9] posits that the mechanisms of synaptic potentiation during wakefulness are directly related to the enhancement of slow wave activity (SWA; 0.5–4.5 Hz) in the electroencephalogram (EEG) during the subsequent sleep. SWA is considered a measure of sleep need, and there is well-established evidence that SWA increases with the time spent awake and progressively decreases during sleep [15].

This hypothesis is based on the observation that increased expression of LTP markers during wakefulness is followed by higher level of SWA during the subsequent sleep [16]. A demonstration that the increase of SWA during sleep depends directly on the LTP mechanisms, and not on wakefulness as such, comes from animal studies showing that when the expression of LTP-related molecules is reduced, due to damages to the noradrenergic system, the SWA peak during sleep is blunted [12, 17, 18]. 

The relation between changes in cortical plasticity during wakefulness and quantitative changes of SWA in subsequent sleep is topographically specific; a higher LTP in a particular cortical area is directly correlated to an increase of the SWA activity in that area. Huber and co-workers [19] have investigated the effects on sleep of a specific visuomotor task (adaptation to a rotated frame of reference) that activates the right parietal cortex [20] and seems to be related to a synaptic potentiation mechanism. Results showed that this task, when compared with a subjectively indistinguishable control task, induced during the subsequent sleep an increase of SWA, which was limited to the right parietal area. This finding supports the hypothesis of the activation of a local homeostatic process during sleep. Similarly, a declarative learning task induced an increase of SWA and spindle activity (12–15 Hz) in the left frontal area during sleep after a training session, which was positively correlated with changes in memory performance [21]. Finally, a potentiation of TMS-evoked EEG responses induced in the premotor cortex, through the application of a 5 Hz repetitive TMS (rTMS), was positively correlated with an increase of SWA in the following sleep, which was topographically specific for the same premotor site [22].

What happens if a synaptic depression process occurs during wakefulness? Huber and co-workers [23] have found that a short-term arm immobilization was associated with impaired motor performance, smaller somatosensory evoked potentials (SEPs), and motor evoked potentials (MEPs) and was followed by a local reduction in SWA during the successive sleep episode. In summary, plastic changes occurring during wakefulness seem to induce coherent and topographically specific local changes in SWA during the subsequent sleep. 

Further support to the hypothesis that changes in cortical plasticity lead to homeostatic modifications in SWA during sleep comes from studies using TMS paired associative stimulation (PAS) protocol, a method that allows to induce plastic changes in the human cortex by coupling, with a fixed interstimulus interval allowing the sensory impulse to reach and energize the S1/M1 cortex, a peripheral electric stimulus and a magnetic pulse on the scalp. The direction of the plastic changes (potentiation or depression) depends on the interval between the stimuli [24–26]. By using the PAS protocol it has been observed that LTP and long-term depression (LTD) lead to an increase and a reduction in SWA during sleep in somatosensory cortex [27], respectively. Other studies have found different, albeit coherent with the general hypothesis, patterns of topographic changes in SWA [28, 29] and spindle activity [28] after the PAS protocol. These findings still support the hypothesis of a link between cortical plasticity and sleep homeostasis but stress the need of a better knowledge of the underlying neurophysiological mechanisms.

In computational studies, it has been observed that stronger synaptic connections are associated with higher SWA [30, 31]. Moreover, an increase in SWA during sleep which is topographically specific for the motor cortex, induced *in vivo *in rats by learning a motor task, was associated with a post-training increase, in the same cortical area, of *c-Fos* and *Arc* levels [32], two activity-dependent proteins involved in motor learning [33–35]. Finally, a causal relation between brain-derived neurotrophic factor (BDNF) expression during wakefulness and subsequent sleep homeostasis has been observed *in vivo*; higher levels of BDNF lead to increased SWA during subsequent sleep [36, 37]. Together, these data suggest that changes in cortical plasticity during wakefulness lead to homeostatic modifications in SWA during sleep, supporting the hypothesis of a direct relation between cortical plasticity and sleep regulation.

## 3. Synaptic Renormalization during Sleep

According to the synaptic homeostasis hypothesis [8, 9] sleep has a functional role in promoting the so-called synaptic downscaling. This process would have the function of restoring the total synaptic strength to a sustainable energy level, favouring memory and performance gain. In other words, the experience-dependent synaptic potentiation occurring during wakefulness is proportionally reflected in the following sleep, and in particular during NREM sleep, a condition which is characterized by slow oscillations and is an ideal *scenario* for the experimental induction of LTD-like mechanisms [38]. SWA would support downscaling by the alternation of a depolarization phase (up-phase) and a hyperpolarization phase (down-phase) [39].

The hypothesis of a link between plastic changes and SWA has received considerable empirical support by the findings of significant fluctuation of different molecular and electrophysiological markers within the sleep-wake cycle. Absolute levels of brain metabolism significantly decrease after a period of sleep [40], consistently with the hypothesis of a synaptic downscaling process during sleep. Moreover, while molecular processes associated with LTP reach lower levels during sleep than during wakefulness [12, 41], molecular changes involved in LTD-like mechanisms increase during sleep, compared with waking time [13, 41]. At an electrophysiological level, animal studies found that different markers of synaptic efficacy increase after prolonged wakefulness and decrease following a period of sleep [41, 42]. 

Notwithstanding this evidence, it is likely that the synaptic homeostasis hypothesis does not allow for a comprehensive understanding of the processes that occur during sleep. Frank [43] points out that different molecules, like *Arc*, observed at high levels in the cerebral cortex after waking, mediate both LTP and LTD processes [13, 44] and the synthesis of GABA in inhibitory interneurons [45, 46]. Their presence, then, is not a clear indication of synaptic potentiation or depression. Moreover, different studies show that some neuromodulators involved in synaptic potentiation increase during NREM sleep [47, 48] and some potentiation-related molecular changes have been observed during sleep [49–52].

These studies seem to question some predictions of the synaptic homeostasis hypothesis in favour of a “consolidation” model, which predicts an increased potentiation of specific neural circuits as a prerequisite of consolidation mechanisms. In this perspective, the consolidation process should involve a sleep-dependent reorganization and redistribution of the newly acquired information between different brain systems, so that it can be defined as a “system consolidation” process [53]. In particular, the parallel activation of specific neocortical and hippocampal networks during wakefulness, which represent, respectively, long-term and temporary store in declarative memory system, should induce a selective reactivation of the same circuits during subsequent sleep [10, 11, 54, 55]. Several findings support this model pointing out to the crucial role played by sleep-specific brain activity in favouring consolidation of memory traces [19, 56–60]. According to the model, slow oscillations originated during SWS in neocortical networks [61] allow the formation of spindle-ripple events that mediates the hippocampus-to-neocortex transfer of memory information. Specifically, the depolarizing up-phase of slow oscillations drives the hippocampus to trigger the reactivation of memory representations that are gradually transferred to the neocortex via thalamo-cortical spindles. In this view, sharp-wave hippocampal ripples represent the reactivation of memory traces while thalamo-cortical spindles, modulating cortical Ca^2+^ influx, provide the background suitable for the changes in neocortical synaptic connections underlying the long-term storing of memory traces in the neocortical respective networks [56–59, 62].

Several evidences in both animals and humans point to the association between learning processes during wakefulness and a coherent neural re-activation during subsequent sleep. Monocular deprivation studies [63, 64] showed that ocular dominance plasticity in cats undergoes a sleep-mediated consolidation process that involves both synaptic potentiation and depression mechanisms (monocular deprivation induces stronger cortical responses to the open eye and weaker responses to the deprived one *after* than *before* sleep). The same pattern of neuronal activation observed during song rehearsal of the Zebra finch was found during sleep [65], and it leads to a temporary deterioration of song quality correlated with a global enhanced learning [66]. A neural re-activation during sleep has been observed in rats after simple spatial tasks [54, 67, 68]. Moreover, the engagement in a learning task results in increased slow oscillations, spindles activity, and hippocampal ripples that seem to be associated with an improved performance [19, 53, 56, 57, 60, 62].

Results from humans studies are consistent with the findings in animals, showing a sleep-dependent re-activation of brain regions involved in previous learning. By positron emission tomography (PET) recordings, Maquet and co-workers [69] found such a specific re-activation during REM sleep that followed a training on a serial reaction time task, while other studies found a re-activation during SWS following declarative learning task [70, 71]. More in detail, Rasch and co-workers [71] showed that the presentation of an odor cue, previously associated with a visual-spatial learning task (memory for cards locations), during sleep induces a greater hippocampal activation, which positively affects task performance. This study provides the first evidence of a causal link between re-activation during sleep and consolidation of memory traces. Interestingly, the enhancement of memory took place only if the odor was presented during SWS, but not during REM, and not in case of a procedural learning task [71]. These findings are consistent with the hypothesis of a differential role of SWS and REM on consolidation of different memory functions [72]. In our view, this dissociation should be taken into consideration in a rehabilitative perspective that takes into account sleep features during treatment. 

Traditionally, according to the “dual processes” theory, the postsleep improvement in procedural memory has been ascribed to REM sleep, while SWS is responsible for the consolidation of declarative memory [73, 74]. Studies using declarative memory tasks showed a better retention performance after sleep if it consists mainly of SWS [21, 71, 73, 75]. In others, boosting SWA enhanced word pairs retention [76–78], but not the retention of procedural memories [76]. Conversely, the recall of a mirror-tracing task improved more after a retention interval spent in REM sleep than in SWS [73], and post-sleep performance improvement in a finger-tapping task was correlated with the time spent in REM sleep [79]. However, this different role for REM and SWS has not been confirmed by other studies. Several nondeclarative tasks, like perceptual discrimination [80] and rotation adaptation [21], are also benefited by SWS, whereas REM sleep in some instances seems to mediate the consolidation of emotional aspects of declarative memory [81]. 

Thus, the “dual processes” theory should be considered an oversimplification, and, actually, a large body of evidence seems to support a different theory, the “sequential” or “double-step” hypothesis [82], which states that consolidation process involves both SWS and REM sleep, regardless of the memory system the traces belong to. According to this hypothesis, what leads to the consolidation of a still labile memory trace during sleep is the repeated pattern of non-REM sleep followed by REM sleep. This view is compatible with the “consolidation” model so that the integration of the two perspectives suggests that consolidation processes would consist in the repetition of sleep cycles with SWS favouring a “system consolidation” of memory by a reactivation and a redistribution of memories to the neocortical “long-term storage.” In this view, subsequent REM sleep allows local processes of consolidation at synaptic level, whereby cortical memory representations are further stabilized [53, 83]. 

With respect to the different role of REM and slow wave sleep (SWS), Chauvette and co-workers [84], using an electrical stimulation at low frequency (1 Hz) of the medial lemniscal fibers in cats, studied the somatosensory cortical-evoked local field power (LFP) responses before and after a period of SWS. The amplitude of the responses in the somatosensory cortex was increased after SWS compared to the previous waking period, suggesting that SWS induces synaptic upscaling, rather than downscaling. On the other hand, the analysis of firing rates across cycles of NREM-REM-NREM sleep in rat hippocampal pyramidal cells and interneurons showed a significant increase in the firing rates during NREM sleep, while a substantial decrease was associated with REM sleep [85]. In addition, the decrease in the firing rates between the first and the last episode of NREM sleep was positively correlated with the amount of theta activity (4–7 Hz) during REM sleep. Together, these findings suggest that different kinds of synaptic renormalization occur during sleep, and the role of REM sleep and theta waves must be better analysed. The results by Grosmark and co-workers could represent a link between the “consolidation” model and the downscaling process. In particular, Born and Feld [86] suggest that a local upscaling of specific memories could occur in concomitance of global downscaling processes. More studies are needed to better understand this intriguing issue. 

## 4. Neural Plasticity and Sleep Deprivation

Since the early empirical observations of De Manacéine [87], it is well known that sleep loss has degrading effects on alertness and performance. If sleep is a behavioral state in which the body recovers physical and mental energies, the lack of sleep can jeopardize the execution of neurocognitive, psychological, and behavioral processes [88].

There are numerous empirical evidences about the harmful consequences of chronic sleep loss, such as drowsiness, reduced alertness, communication difficulties, and cognitive deficits [89, 90]. In particular, different forms of learning are negatively affected by sleep deprivation in humans and animals [5–7], albeit recent findings point out that long-term consolidation does not seem to be affected by sleep loss in adolescents [91].

Memory consolidation is impaired also in different clinical samples characterized by disturbed sleep. Patients with primary insomnia showed a decreased sleep-dependent memory consolidation in procedural and declarative learning associated with the reduction of REM sleep [92, 93] and SWS [94], respectively. Moreover, most of neurodegenerative diseases, like Alzheimer's disease (AD), Parkinson's disease (PD), or dementia with Lewy bodies (DLB), which are usually characterized by memory impairment, share a common pattern of sleep features. In these diseases, sleep is usually more fragmented, SWS is decreased, and spindles, K-complexes and REM sleep are often reduced (for a review, see [95]). 

The detrimental effects of sleep loss on memory suggest a deterioration of the underlying neuronal processes. In particular, alterations of LTP/LTD mechanisms may underlie at least a part of the behavioural alterations observed during sustained wakefulness. 

One of the most well-established consequences of sleep deprivation is the increase of delta and theta EEG activity [96, 97], mainly in frontal cortical areas [98–100], interpreted as an index of higher “recovery need” [98]. It has been proposed that experience-dependent plasticity is directly linked to local changes in the electrophysiological expression of sleep need, as indexed by increased SWA after prolonged wakefulness [8, 9].

An intriguing question is whether and how prolonged periods of wakefulness can affect plastic processes. Different *in vitro *studies show that sleep loss inhibits LTP in the hippocampus but enhances LTD mechanisms [101–103]. This suggests that the induction of LTP could be saturated after sleep deprivation. More recently, it has been observed in cat and mice cortical slices that the amplitude and frequency of miniature excitatory postsynaptic currents increase after wakefulness and decrease after sleep [42]. Moreover, an increase in number and size of central synapses was observed in *Drosophila melanogaster* after prolonged wakefulness, and a subsequent decrease was possible only after sleep [104]. In this study, qualitative characteristics of wakefulness were also evaluated; richer experiences during wake were followed not only by a higher sleep need, but also by a greater synaptic growth. 

Vyazovskiy and co-workers [41] found that synaptic strength, in terms of amplitude and slope of local field power (LFP), increases after a period of sustained wakefulness in rats, and that the induction of LTP is easier after sleep rather then after a waking period. Furthermore, cortical neurons fire at higher frequency [105] and cortical excitability is increased [106] after sleep deprivation.

At molecular level, these changes involve the delivery of postsynaptic glutamatergic AMPA receptors (AMPARs) containing GluR1 subunit [41]. In particular, GluR1-containing AMPAR levels increase with time spent awake and decrease during sleep [41]. These morphological and functional changes are likely connected with the variations in the firing rates of cortical and hippocampal neurons recently described for wake-sleep cycle and sleep deprivation in rats [105]. Furthermore, high levels of different pre- and postsynaptic proteins and proteins involved in neurotransmitters release have been found in *Drosophila melanogaster* after sleep deprivation, while their levels were low after sleep [107].

Considering that brain metabolism accounts for 20% of all the body rest metabolism [108] and that around 75% of brain's energy consumption is due to glutamatergic synaptic signalling (action potentials, post-synaptic potentials, and repolarization) [109], it is reasonable to assume that a widespread cortical increase of firing rate during wakefulness combined with a rapid and progressive increase of cortical extracellular glutamate levels [110] results in a raising of brain metabolic costs. In contrast, NREM sleep, characterized by low firing rate [105] and low cortical extracellular glutamate levels [110], is associated with a reduction in energy demand. 

Taken together, these findings suggest that synaptic strength progressively increases with time awake, leading to high energy costs and saturating learning process. Sleep seems to be necessary for a homeostatic renormalization of cortical synapses, but it remains unclear how sleep propensity is accumulated during wakefulness. In recent years, adenosine assumed increasing importance as mediator of connection between brain activity during wake and sleep regulation, whereas basal forebrain seems to be the “adenosine sensor” of the brain responsible for the adenosinergic modulation of sleep-wake states (for review see [111]). In brief, adenosine, through its action on A1 receptor, promotes the transition from wakefulness to SWS by inhibiting wake-active neurons in basal forebrain, which are connected to cortical regions. Sleep begins when the activity of the wave-active cells decreases sufficiently. During sleep, neuronal activity decreases causing a reduction of extracellular adenosine concentration. A sufficient reduction of extracellular adenosine makes wake-active cell in basal forebrain free from adenosine inhibition resulting in the initiation of a new waking period [111]. Consistent with this hypothesis, an experimentally induced energy depletion by 2,4-dinitrophenol (DNP, molecule which prevents the synthesis of ATP) infusions in basal forebrain (but not outside) increases subsequent sleep need [112]. 

Taken as a whole, results from animal studies suggest that sleep deprivation has detrimental effects on synaptic plasticity. However, it should be borne in mind that processes like neuronal firing, metabolic activity, and synaptic potentiation represent different aspects of neuronal functioning, and the relationship between them is not simple, although the state of knowledge does not provide a complete unifying framework for these different aspects of neural functioning yet.

At present, there is no direct evidence in humans about the modification of plastic processes after sleep loss. Nevertheless, changes in cortical excitability have been widely investigated (mainly by means of TMS) but results are not univocal. Many studies point to an increase of cortical excitability during prolonged wakefulness in healthy subjects, in terms of modulation of motor evoked potentials [99, 113], and TMS-evoked potentials [114]. It should be remembered that an increase of cortical excitability after prolonged wakefulness is usually observed in epileptic patients [115, 116] and that sleep loss increases seizures [117]. On the other hand, other studies were not able to provide any evidence of a modulation of sleep deprivation on cortical excitability [118, 119] or found conflicting results [120]. Although such negative findings can be explained by a lack of statistical power due to the small sample size of these last studies, the mechanisms underlying changes in human cortical excitability in condition of sleep loss remain unclear. Moreover, in most cases only changes in frontal and prefrontal cortical excitability have been investigated. The only study that assessed how sleep deprivation modulates the responsivity of the somatosensory cortex showed an increase in early SEPs components amplitude, but it did not account for the potential influence of circadian factors [121]. 

## 5. The Role of Sleep-Dependent Plasticity in Motor and Cognitive Rehabilitation

As discussed in the previous paragraphs, we still miss a complete understanding of the plastic processes occurring during sleep. Nevertheless, a coherent pattern of empirical findings shows that plastic changes during wake can affect subsequent sleep, which in turn has a beneficial effect on plastic mechanisms and learning processes. So, the existence of a link between sleep and synaptic plasticity is widely accepted. 

Since plastic processes are involved in functional recovery from different neuropsychological disorders and after brain damage, the understanding of how SWA during sleep can affect rehabilitation-dependent plasticity seems a suggestive issue. If sleep has a role in modulating cortical plasticity, rehabilitative protocols should be designed considering how sleep could improve recovery. In the present section, we will consider the possible role of sleep-dependent synaptic plasticity in the rehabilitation from different neuropsychological conditions.

### 5.1. Stroke

Animal models show that ischemic stroke can induce a SWS increase and a paradoxical sleep decrease in mice [122] and rats [123]. Mice undergoing treatment with *γ*-hydroxybutyrate (GHB), a drug used to promote SWS in humans [124], showed a faster recovery of the grip strength in the paretic forelimb, when compared with those treated with vehicle saline [125]. Moreover, sleep disturbance and sleep disruption negatively affect poststroke recovery in rats [123, 126] impairing axonal sprouting and neurogenesis [126], two cellular processes associated with functional recovery [127–129].

Only few human studies on this issue are available at the moment, but they show promising results. Several experiments have been designed to understand if sleep can affect motor learning in post-stroke patients [130–132], based on the observation of a sleep-dependent memory consolidation of different motor tasks in healthy subjects [133, 134]. Results showed that sleep enhances offline implicit and explicit motor learning of a continuous sequencing task [130, 131] and improves spatial tracking accuracy and anticipation of upcoming movements during a continuous tracking task [132]. Sleep seems to induce a selective enhancement in sequential motor learning and performance also in patients with prefrontal lesions, with no improvement in verbal and working memory [135]. A limitation of these studies is the absence of EEG recordings, so hypotheses on the electrophysiological basis of the influence of sleep on motor performance in post-stroke patients remain at a speculative level. Nevertheless, these findings suggest that an appropriate management of sleep-wake cycle in these patients could promote motor recovery. Siengsukon and Boyd [136] stated that sleep between therapy sessions should be supported, with the aim to support off-line learning, and a quiet environment for sleep should be ensured to patients. Moreover, post-stroke patients are often characterized by different sleep disorders like hypersomnia, insomnia, sleep-related breathing disturbances, or restless legs syndrome (for a review, see [137]), and other factors like depression or side-effects of pharmacological treatments could negatively affect sleep in these patients. The importance of managing these factors for the rehabilitation outcome should not be underestimated.

### 5.2. Obstructive Sleep Apnea

Obstructive sleep apnea (OSA) is a common breathing and sleep disorder, characterized by cessations or reduction in respiration due to pharyngeal collapse during sleep that induce intermittent hypoxia and sleep fragmentation [138, 139] increasing daytime sleepiness [139] and risk for cardiovascular disease [140]. OSA is associated with neurocognitive impairment, with negative influence on vigilance, attention, executive functioning and memory [141, 142]. Nevertheless, the neural basis of cognitive impairment in OSA patients is not well understood yet. Animal models show that hypoxia induces apoptosis in cortical and hippocampal neurons [143], but neural functioning may be already compromised before the beginning of the apoptotic process [144]. Results from recent studies suggest that changes in synaptic plasticity could account for cognitive impairment in OSA patients [144, 145]. In fact, Xie and co-workers [145] have found that chronic intermittent hypoxia in mice induced an impairment in hippocampal early and late-phase LTP and a reduction of the expression of the brain-derived neurotrophic factor (BDNF), a neurotrophin that modulates synaptic plasticity [146, 147]. Moreover, the increase in oxygen and nutrient demand induced by chronic intermittent hypoxia should lead to adaptive homeostatic changes in the blood-brain barrier that, at long-term, could influence brain's microenvironment, inducing an impairment in plastic processes and cognitive performance [148]. A better understanding of the plastic changes occurring in OSA patients, as well as their possible role in cognitive impairment, is of great importance at a therapeutic level. At present, albeit many studies show that the continuous positive airway pressure (CPAP), the treatment of choice for OSA, improves different cognitive functions [149, 150], a recent meta-analysis points out that only a small recovery in the attention domain can be observed after CPAP treatment [151]. Therapeutic strategies based on the enhancement of synaptic plasticity may be useful to improve neurocognitive functioning in OSA patients, particularly for what concerns memory consolidation. For example, treatment with multiple intraventricular injections of BDNF in mice has beneficial effects on the LTP impairment induced by hypoxia [145] and has been proposed as a possible method to reduce neurocognitive impairments [144]. 

### 5.3. Alzheimer's Disease

Sleep in patients affected by Alzheimer's disease (AD) is characterized by a general accentuation of the sleep modifications which are observed in normal aging [152]: sleep fragmentation, decreased SWS and REM sleep, increased percentage of stage 1, alterations of sleep spindles, and K-complex [153–155]. Moreover, different studies have found an EEG slowing during a condition of resting wakefulness in AD and Mild Cognitive Impairment (MCI) patients [156–161]. A similar phenomenon of EEG slowing in these patients has been observed also during REM sleep, particularly in temporoparietal and frontal sites, with increased delta and theta frequencies and reduced alpha and beta activity, as compared with normal elderly [162, 163]. Recently, melatonin treatment for the management of sleep has been proposed as a possible therapeutic strategy in AD patients, and many studies have shown its beneficial effects on sleep quality, depressive symptoms, and neuropsychological performance in MCI patients (for review see [164]). Albeit the mechanisms underlying the beneficial melatonin effects in AD and MCI remain unclear, Kang and co-workers [165] have found in mice that sleep reduces synaptic anomalies associated with amyloid precursor protein (APP), one of the typical synaptic alterations observed in AD [166]. These data raise the possibility that the positive effects of sleep in AD and MCI are associated with an enhancement of synaptic plasticity. Since plastic processes are strongly impaired in AD patients [167], a reduction of sleep alterations could be useful to restore synaptic plasticity and to limit or to slow down the cognitive decline in such patients. Future studies should be designed to understand if synaptic plasticity in AD patients benefits from the restoration of specific sleep features (i.e., SWS) or if a general improvement of sleep quality is needed.

### 5.4. Autism

Prevalence of sleep disorders in children with autism ranges from 40% to 80% [168–170]. Sleep in autistic children is characterized by long sleep latency, nocturnal awakenings, short sleep duration, low sleep efficiency, circadian rhythm disturbances, increased REM density and stage 1 sleep, reduction of REM sleep and SWS, and decreased spindle activity (for review see [171]). Moreover, behavioural insomnia syndromes and REM sleep behaviour disorder have been often observed [172].

Different studies have found a deficit in melatonin secretion in autistic patients [173–175] that seems to represent a risk factor (and not a consequence) of autism [176]. Recently it has been proposed that learning disabilities in autisms are related to an abnormally high LTP linked with pineal hypofunction, low serum melatonin levels, and sleep dysfunction [177]. According to the authors, promoting sleep by means of a melatonin treatment may reduce learning disabilities by restoring the synaptic plasticity. Melatonin treatment improves sleep quality in autistic patients [178, 179] and secretin, a hormone that stimulates melatonin [180], induces a temporary improvement of autism symptoms [181, 182]. 

## 6. Sleep-Dependent Plasticity and Rehabilitation: Future Directions

The promotion of sleep between therapy sessions, the prompt treatment of associated sleep disorders, and the improvement of sleep quality by adequate environment and melatonin administration could be considered as general indications to support the beneficial effects of sleep on synaptic plasticity in different clinical conditions. However, future studies should be designed to develop methods that directly enhance sleep-dependent plasticity, in order to optimize its role in functional recovery. Massimini and co-workers [183] have found that TMS at a frequency of <1 Hz applied during NREM sleep triggers slow waves in humans. It would be interesting to understand if this kind of stimulation has a fallout on memory performance. Recently, it has been observed that the application of a weak electric anodal current at 0.75 Hz (the frequency of the sleep slow oscillations in humans) during NREM sleep induces an increase in slow oscillations and spindle activity and facilitates declarative memory consolidation [77]. Conversely, transcranial direct current stimulation (tDCS) at 5 Hz (theta frequency) during NREM sleep provokes a general decrease in slow oscillations, a frontal reduction of slow EEG spindle power, and decreases declarative memory consolidation, as well as increases gamma activity (25–45 Hz) when applied during REM sleep [184]. Slow oscillations and spindle activity can be enhanced also by an auditory stimulation in phase with the ongoing oscillatory EEG activity (auditory closed-loop stimulation) during sleep, again with beneficial effects on declarative memory consolidation [78]. These findings suggest that we can influence subsequent memory performance by modulating different EEG rhythms during sleep, probably affecting synaptic plasticity [77]. Future studies should investigate the plastic process underlying the effect of transcranial stimulation and auditory closed-loop stimulation during sleep on memory, and if such different kinds of stimulations can have long-term beneficial effects on memory performance in patients affected by different clinical conditions characterized by memory impairment. Similar questions arise for what concerns the possible clinical usefulness of the previously-quoted (see Section 3) phenomenon of memory consolidation improvement induced by reexposure to odor cues during SWS [71].

An implication of the link between sleep and plasticity is that monitoring SWA during sleep in patients undergoing a rehabilitative treatment may represent a useful method to better understand how that specific rehabilitative protocol works. For example, Sarasso and co-workers [185] have recently used the quantitative analysis of sleep high-density EEG, in post-left hemisphere stroke patients with nonfluent aphasia, aimed to assess plastic changes induced by the Intensive Mouth Imitation and Talking for Aphasia Therapeutic Effects (IMITATE), a computer-based therapy for post-stroke aphasia rehabilitation [186]. Results showed that a single intensive IMITATE session can induce a SWA increase during the initial 30 minutes of the first NREM sleep cycle over the left premotor and inferior parietal areas. A SWA increase was also observed in both left and right frontal areas, but with a peak at the right sites. At a behavioural level, IMITATE induced an improvement in language skills, assessed by the Western Aphasia Battery Repetition Scale [187]. Albeit these are only very preliminary results (only 4 patients), they are indicative of the possible applications of the sleep EEG topography analysis for the assessment of plastic changes induced by a rehabilitation protocol, opening new perspectives for the comprehension of the neurophysiological basis of rehabilitation. 

## 7. Conclusions

The nature of the link between sleep and synaptic plasticity is not completely definite. Different processes of synaptic renormalization seem to occur during sleep, but the definition of their functional role needs further investigation. Nevertheless, sleep and synaptic plasticity seem to be strongly related. The induction of plastic changes during wake produces coherent and topographically specific local changes in SWA during the subsequent sleep. Moreover, sleep seems to restore synaptic plasticity, with beneficial effects on learning processes, while sleep deprivation induces alteration in LTP/LTD mechanisms, increases cortical excitability, and has negative consequence on learning. Growing evidence suggests that promoting sleep may be useful to restore synaptic plasticity in different pathological conditions. Since plastic processes are essential for functional recovery, a management of sleep-wake cycle in patients and an adequate treatment of associated sleep disturbances could be crucial for the rehabilitation outcome. Moreover, different methods like TMS, tDCS, auditory closed-loop stimulation, and odor cues, applied during sleep, can promote memory performance, probably enhancing synaptic plasticity. Future studies should test the possibility to use such techniques to support functional recovery in neuropsychological patients. Finally, monitoring SWA during sleep may help to understand how a rehabilitative protocol affects plastic mechanisms. 
